# An Update on the Structure of hERG

**DOI:** 10.3389/fphar.2019.01572

**Published:** 2020-01-24

**Authors:** Andrew Butler, Matthew V. Helliwell, Yihong Zhang, Jules C. Hancox, Christopher E. Dempsey

**Affiliations:** ^1^School of Physiology, Pharmacology and Neuroscience, Medical Sciences Building, Bristol, United Kingdom; ^2^School of Biochemistry, Medical Sciences Building, Bristol, United Kingdom

**Keywords:** hERG, cryo-EM structure, C-type inactivation, drug block, KCNH, long QT syndrome, short QT syndrome, channelopathy

## Abstract

The human voltage-sensitive K^+^ channel hERG plays a fundamental role in cardiac action potential repolarization, effectively controlling the QT interval of the electrocardiogram. Inherited loss- or gain-of-function mutations in hERG can result in dangerous “long” (LQTS) or “short” QT syndromes (SQTS), respectively, and the anomalous susceptibility of hERG to block by a diverse range of drugs underlies an acquired LQTS. A recent open channel cryo-EM structure of hERG should greatly advance understanding of the molecular basis of hERG channelopathies and drug-induced LQTS. Here we describe an update of recent research that addresses the nature of the particular gated state of hERG captured in the new structure, and the insight afforded by the structure into the molecular basis for high affinity drug block of hERG, the binding of hERG activators and the molecular basis of hERG's peculiar gating properties. Interpretation of the pharmacology of natural SQTS mutants in the context of the structure is a promising approach to understanding the molecular basis of hERG inactivation, and the structure suggests how voltage-dependent changes in the membrane domain may be transmitted to an extracellular “turret” to effect inactivation through aromatic side chain motifs that are conserved throughout the KCNH family of channels.

## The Significance of the hERG Potassium Channel

The *KCNH2* gene encodes a voltage sensitive potassium (K^+^) channel protein, hERG1 (“hERG” for simplicity), which mediates rapid delayed rectifier K^+^ current (I_Kr_) that makes a major contribution to the repolarization phase of cardiac action potentials, effectively controlling the action potential duration (APD) and QT interval observed in electrocardiograms (Sanguinetti and Tristani-Firouzi, 2006; Vandenberg et al., 2012). This cardiac function of hERG is a property of its unique gating characteristics: like other voltage-sensitive K^+^ channels, hERG opens following membrane depolarization as a result of voltage-dependent responses of its voltage sensor domain; however the channel almost immediately inactivates, limiting K^+^ passage until the start of the repolarization phase of the AP. In addition to the rapid onset and recovery from inactivation, hERG deactivates very slowly so that outward K^+^ current is passed even as the membrane potential returns toward the resting potential. This strongly supports efficient repolarization of the cardiac AP. The gating kinetics of hERG also enable the channel to generate rapid transient currents late in action potential repolarization/early diastole, to protect against arrhythmogenic premature depolarizations (Lu et al., 2001). Additionally, the deactivation kinetics of the channel allow I_Kr_ to influence diastolic depolarization of cardiac pacemaker cells (Ono and Ito, 1995; Mitcheson and Hancox, 1999). Inherited mutations in hERG that attenuate inactivation (“gain of function”) result in premature repolarization and shortening of the QT interval (short QT syndrome; SQTS) (Campuzano et al., 2019; Hancox et al., 2019). Loss of function mutations, many (but not all) of which arise from disrupted trafficking of hERG to the cell surface (Anderson et al., 2014), can result in inefficient repolarization and thus an elongation of the QT interval (long QT syndrome; LQTS). Each of these may result in cardiac arrhythmias.

At least as important as its role in hERG-associated congenital arrhythmias is the pharmacological susceptibility of hERG to block by a variety of functionally- and structurally-diverse drugs which underlies the drug-induced form of *acquired* LQTS with a susceptibility to *Torsades de Pointes* (TdP) (Vandenberg et al., 2001; Sanguinetti and Tristani-Firouzi, 2006; He et al., 2013; Kalyaanamoorthy and Barakat, 2018). The potential for involvement of hERG in drug-related arrhythmia is sufficiently strong that existing preclinical guidelines require testing of all new drugs for hERG block, typically using a hERG assay (Hancox et al., 2008). Understanding the molecular basis for promiscuous drug block of hERG would be enormously beneficial in efforts to pre-screen drugs for hERG liability in drug development programs, and to reduce adverse effects in otherwise-useful drugs through targeted chemical modification. Likewise, insight into the molecular basis for hERG's anomalous gating properties, particularly the mechanisms of rapid onset and recovery from inactivation, as well the perturbation of inactivation in congenital short QT mutations, should greatly facilitate development of therapeutic interventions for SQTS (Hancox et al., 2018).

Considerable effort has been made to understand the molecular basis of hERG's unique gating kinetics and susceptibility to pharmacological inhibition. In the long absence of a hERG structure, much of the functional data on wild type hERG and channel mutants has been interpreted using homology models of the channel (Villoutreix and Taboureau, 2015). In this light, a recent cryo-EM structure for hERG (Wang and MacKinnon, 2017) is very welcome, providing the potential to facilitate major advances in understanding hERG drug block and the molecular basis for hERG channel gating. This short review provides an update of progress in these areas some two years after the publication of the structure, focusing on the membrane domain of the channel that is most relevant to hERG pharmacology. A recent review contains a good overview of the structure of hERG's cytoplasmic domains (Robertson and Morais-Cabral, 2019).

## The hERG Structure and Drug Block

**Figure 1:**
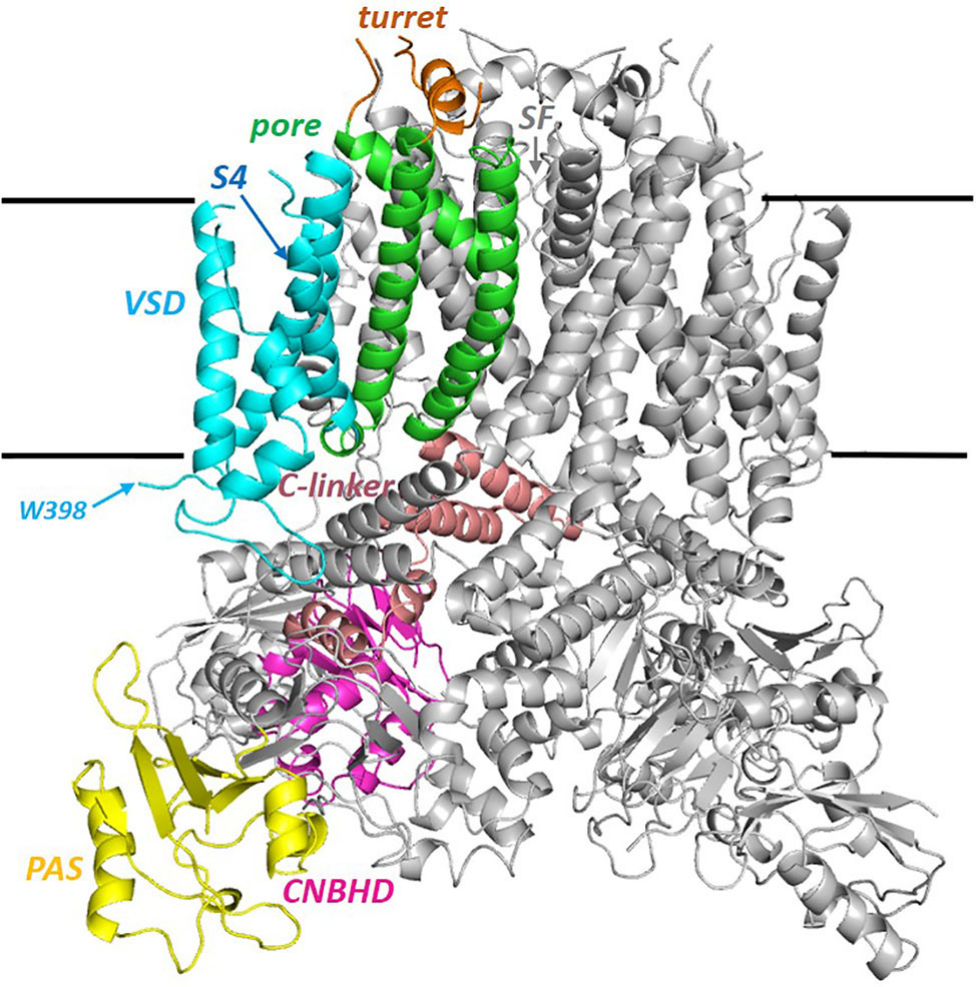
Side view of the full hERG cryo-EM structure (PDB: 5VA2; hERG_T_) with one of the four subunits colored by domain. PAS (the N-terminal Per-ARNT-Sim) domain; the cytoplasmic C-linker links the S6 helix of the pore domain (green) to the CNBHD (Cyclic Nucleotide Binding Homology Domain). VSD (Voltage Sensor Domain). W398 is the first residue of the membrane domain of hERG for which atom density is defined. Sequence breaks in some extra extracellular loops are regions lacking atom density in the cryo-EM structure. The horizontal black lines mark the approximate limits of the non-polar part of the bilayer membrane.

**Figure 2:**
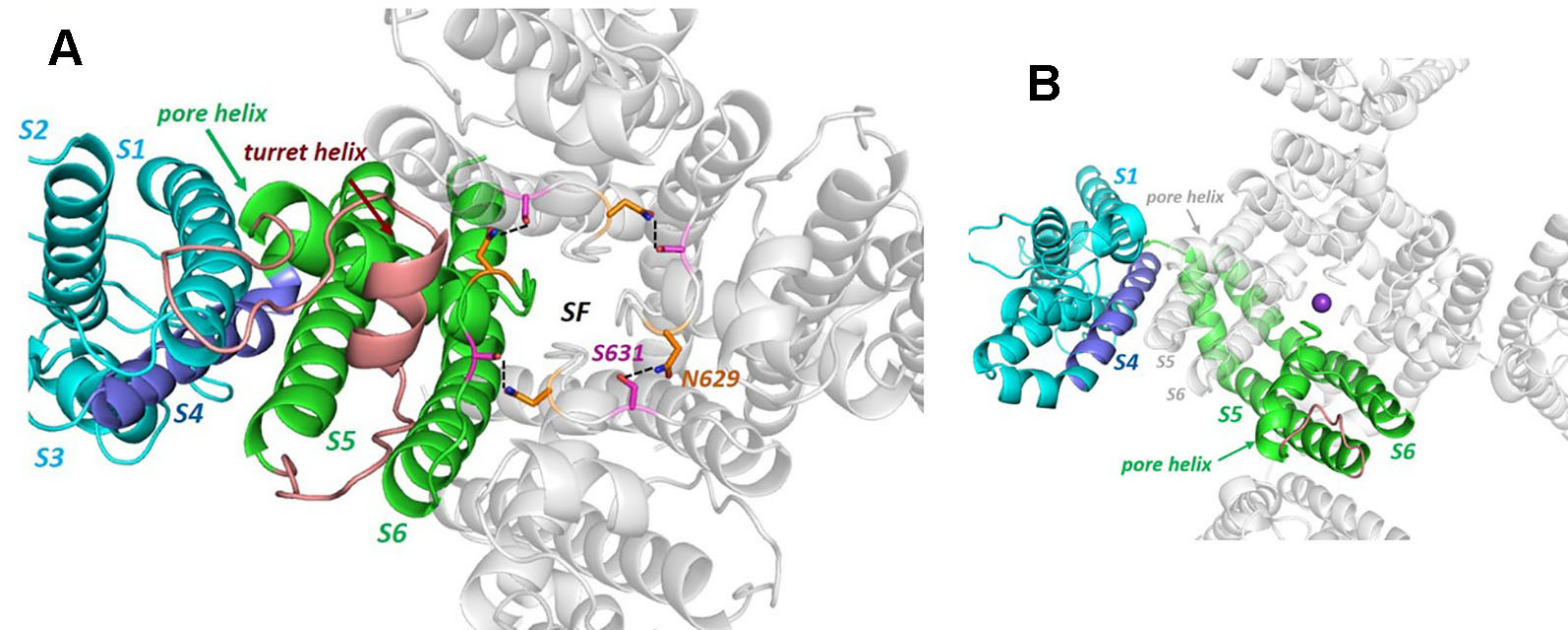
**(A)** The hERG cryo-EM structure viewed from the extracellular side of the membrane illustrating the non-domain-swapped subunit organization in which the voltage sensor domain (VSD) is packed against the pore domain of the same subunit. The long turret sequence containing a turret helix, that links the top of S5 with the N-terminal end of the pore helix, is colored brown (some atom density in the non-helical region of the turret is missing in the cryo-EM structure and is modelled into the structure shown). Polar side chains of N629 and S631 form a hydrogen-bonded ring that links subunits around the top of the selectivity filter. **(B)** Equivalent view of the Kv1.2/2.1 K^+^ channel chimera structure [K_v_Chim; PDB: 2R9R (Long et al., 2007)] illustrating domain-swapping and a very short “turret” sequence (brown). Although the VSDs are packed against pore domains of adjacent subunits in domain-swapped channels, the relative *inter*subunit juxtaposition of S5 and the pore helix with the VSD is similar to the *intra*subunit juxtaposition of VSD and S5 and pore helix in hERG (and rEAG). The purple sphere is a K^+^ ion in the S1 position of the selectivity filter.

The hERG cryo-EM structure (PDB:5VA2; **Figure 1**) was obtained using a truncated construct (hERG_T_) in which cytoplasmic domain residues 141 to 350 and 871 to 1,005 were removed from the full length 1,159 residue protein to suppress aggregation that occurs with wild type hERG (Wang and MacKinnon, 2017). The first deletion removes most of the linker region that connects the N-terminal Per-ARNT-Sim (PAS) domain with the voltage sensor (VSD) domain (the VSD S1 helix starts near P405, a short distance C-terminal to W398, the first amino acid of the membrane domain visible in the cryoEM structure; **Figure 1**). The second deletion eliminates much of the long cytoplasmic C-terminal tail that follows the cytoplasmic cyclic nucleotide binding homology domain (CNBHD) that extends to near residue R863. Otherwise the membrane domain of the channel is fully contained within the hERG_T_ construct, and this undergoes voltage-dependent activation, fast inactivation, recovery from inactivation and slow deactivation with properties much like WT hERG with only a small (~5 mV) positive shift in the voltage-dependence of inactivation. A second truncated construct (hERG_TS_; PDB:5VA1) with residues 141–380 and 871–1,005 deleted, served as a background for an S631A mutation (hERG_TS_ S631A; PDB:5VA3) that, like wild type hERG S631A, has attenuated inactivation (see **Figure 2** for the location of S631). hERG_TS_ has a voltage dependence of activation shifted around 20 mV to negative potentials but hERG_TS_ S631A shows attenuated inactivation relative to hERG_TS_ analogous to the attenuation of inactivation in hERG S631A relative to hERG, as described later.

**Figure 3:**
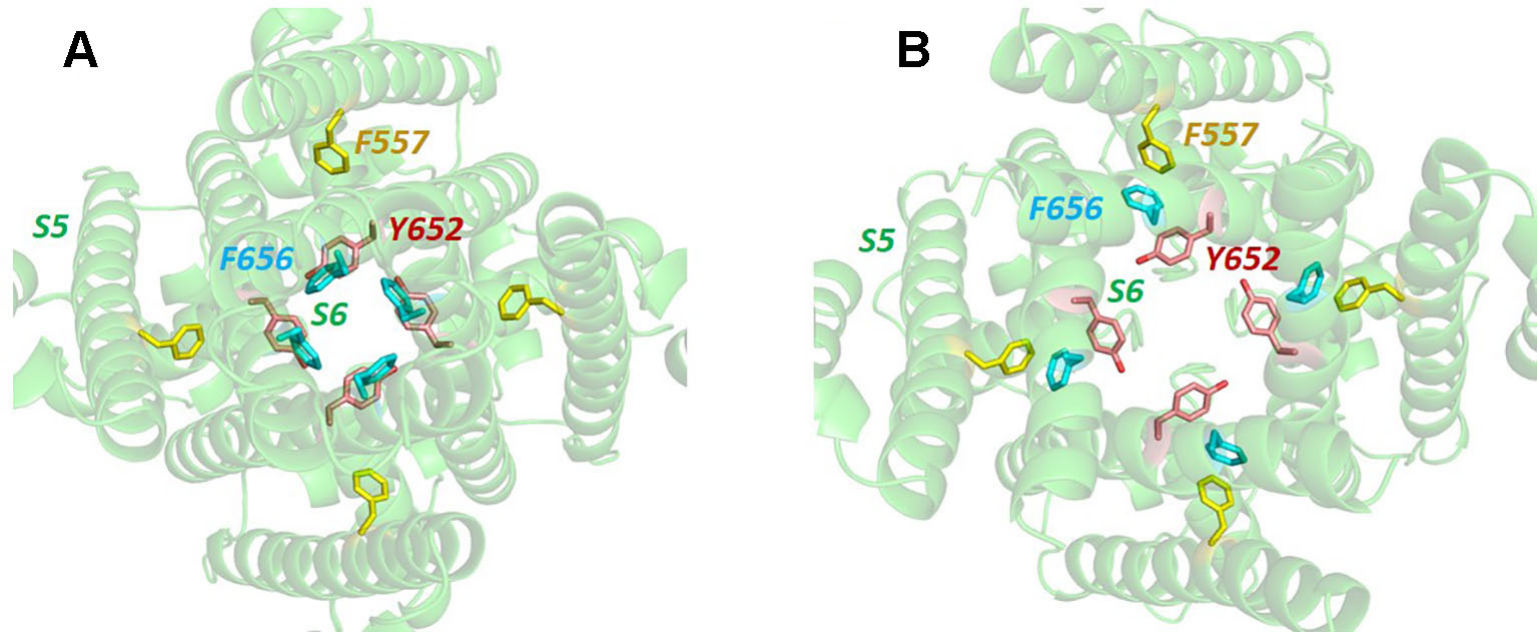
**(A)** Bottom up view of the pore domain of a hERG homology model built on the structure of the highly homologous rEAG structure [PDB: 5K7L (Whicher and MacKinnon, 2016)] which has an activated voltage sensor but a closed pore. In this model the side chains of key amino acids for hERG channel block, Y652 and F656, are oriented towards the K^+^ permeation pathway at the centre of the pore. **(B)** In the open pore hERG cryo-EM structure, the F656 side chains are oriented away from the pore center towards the F557 side chain on the S5 helix.

**Figure 4:**
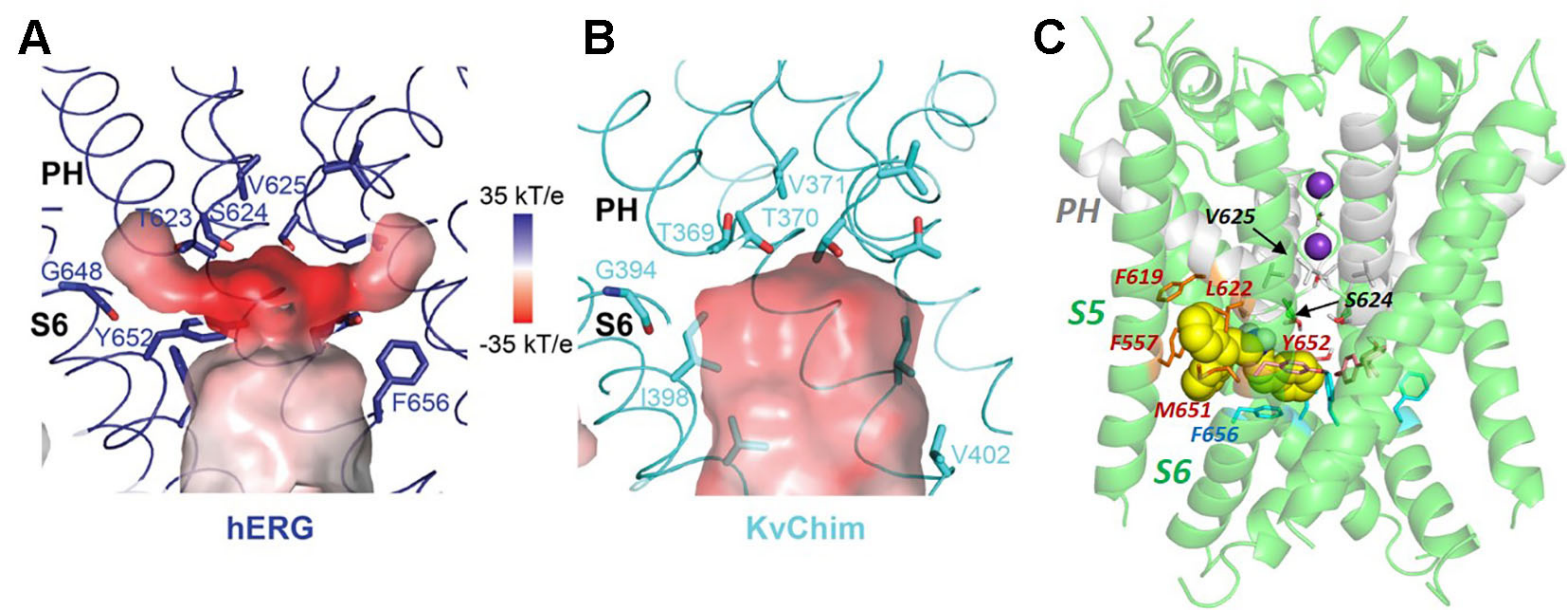
Comparison of the hERG pore cavity **(A)** with that of the K_v_1.2/2.1 channel chimera (K_v_Chim) **(B)**. The hERG cavity is smaller than the equivalent K_v_Chim cavity and has hydrophobic “pockets” that project from the central cavity below the bottom of the selectivity filter and underneath the pore helix (PH). The pore helix negative dipole charges focus a strong negative electrostatic potential below the selectivity filter which contributes to the binding energy for positively-charged hERG pore blockers. **(C)** The hERG blocker “Cavalli-2” [(Cavalli et al., 2012); yellow space filling representation] can be docked partially within a hydrophobic pocket although readjustment of F656 side chains is required for interaction of blocker with more than one F656 side chain [see text; adapted from (Helliwell et al., 2018)]. Panels **(A** and **B)** from (Wang and MacKinnon, 2017) with permission.

What would formerly have been a surprising aspect of the hERG structure, the absence of domain swapping amongst the four subunits of the tetrameric membrane domain found in other voltage-sensitive K^+^ channels (**Figure 2**), turns out not to be so surprising since we were primed with a non-domain-swapped structure of a related protein, EAG, published by Whicher and MacKinnon in 2016 (Whicher and MacKinnon, 2016). The high homology between hERG and EAG has allowed homology models of hERG to be built on the EAG structural template [e.g. (Butler et al., 2019)], and these suggested that two key amino acid side chains in the channel pore domain known to be important for drug block, Y652 and F656, likely project towards the K^+^ permeation pathway to interact with hERG-blocking drugs that diffuse into the pore when the channel opens from the cytoplasmic side of the membrane (**Figure 3A**). It was a surprise, therefore, to observe in the hERG open pore structure that the F656 side chains project *away* from the K^+^ permeation pathway towards the outer pore helix (S5; **Figure 3B**). Since mutation of F656 has the largest effect in attenuating drug block by a number of hERG blockers (Witchel et al., 2004; Kamiya et al., 2006; Du et al., 2014; Melgari et al., 2015a; Helliwell et al., 2018) (see **Table 1**), the expectation was that these blockers interact with more than one F656 side chain, as observed in computational docking with a variety of hERG pore models (e.g. (Farid et al., 2006; Stansfeld et al., 2007; Stary et al., 2010; Dempsey et al., 2014). This interpretation seems to be incompatible with the arrangement of F656 side chains in the cryoEM structure (Helliwell et al., 2018). On the other hand, Wang and MacKinnon identified hydrophobic “pockets” that project away from the central pore cavity below the selectivity filter and pore helix (**Figure 4**) (Wang and MacKinnon, 2017); these pockets provide potential interaction sites for hERG blockers and there is no question that in computational docking studies many well characterized hERG blockers can be biased to bind partially within these pockets (e.g. **Figure 4C**). However, when bound within a hydrophobic pocket, drugs are able to interact with only one F656 side chain, contrary to the expectations described above.

**Table 1:** Fold-change in IC_50_ relative to WT IC_50_ from alanine mutagenesis of hERG pore residues for selected hERG blockers.

hERG blocker	Cell line	Temp.	WT IC_50_ (nM)	Fold Increase in IC_50_ (mutant IC_50_/WT IC_50_)							Ref
				Pore helix		SF	S6				
				T623A	S624A	V625A	G648A	Y652A	F656A	V659A	
**amiodarone**	HEK293	37°C	45	6.5	22	6 (E)	5.7	20	17	9.9	(Zhang et al., 2016)
**Cavalli-2**	HEK293	37°C	36	16	7			17	75		(Helliwell et al., 2018)
**clomipramine**	HEK293 Oocyte	36 °C room temp.	130 12,400					6	12		(Jo et al., 2008)
**cisapride**	Oocyte	room temp.	133			2	1	100	40		(Mitcheson et al., 2000)
**clofilium**	Oocyte	room temp.	30	12	381	250		1329	484		(Perry et al., 2004; Perry et al., 2006)
**dofetilide** (estimated)	Oocyte	room temp.	420	7	9	130	171	25	62	3	(Kamiya et al., 2006)
**E-4031** (estimated)	Oocyte	room temp.	570	4	13	86	40	31	89	4	(Kamiya et al., 2006)
**flecainide**	HEK293	37 °C	1,490	5.6	1.8	27.5	8.9	3.4	141.5		(Melgari et al., 2015a)
**ibutilide**	Oocyte	room temp.	28	54	93	>300	140 (E)	67 (E)	140 (E)	18 (E)	(Perry et al., 2004)
**MK-499**	Oocyte	room temp.	34	5		54	55	94	650		(Mitcheson et al., 2000)
**ranolazine**	HEK293	37°C	8,030	19	8	8		22	53		(Du et al., 2014)
***R*-roscovitine**	Oocyte	room temp.	196,000	5.4	0.8			2.9	42		(Cernuda et al., 2019)
**terfenadine**	Oocyte	room temp.	134			1.5	1.5	150	100		(Mitcheson et al., 2000)
**verapamil**	HEK293 Oocyte	room temp.	143 5,100					16	20		(Zhang et al., 1999; Duan et al., 2007)
**ziprasidone**	HEK293 Oocyte	37 °C room temp.	120 2,800					140	357		(Su et al., 2006)

(E), estimated. The data report the effects on drug block of hERG mutations in pore helix residues, V625 in the selectivity filter (SF) or residues on the S6 helix. The fold increase in IC_50_ relative to its WT control is given as IC_50_ mutant/IC_50_ WT. Estimated fold increases in IC_50_ are tabled where experimentally derived IC_50_ values were not reported; the estimated values were calculated using available single dose data, by using a standard Hill equation: Fractional block = 1/(1 + (IC_50_/[drug])^h^), and assuming h = 1. Table updated from (Zhang et al., 2016).

There are a couple of ways in which an apparent disparity between experimental mutagenesis studies and the hERG structure might be resolved. Firstly, the interpretation of multiple F656 side chain interactions for blockers with high F656-dependence might be mistaken and some of the high F656 dependence might relate to allosteric contributions of F656 to the structure of the drug binding site. It has been suggested, for example, that F656 might stabilize conformations of the Y652 side chain that promote strong interactions with blockers (Vaz et al., 2017). However for several drugs, mutation of F656 produces much stronger attenuation of block than does mutation of Y652 (see **Table 1**); block of hERG by flecainide, for example, is relatively insensitive to mutation of Y652 to alanine, whereas block is attenuated by nearly 150-fold in the F656A mutant (Melgari et al., 2015a). Likewise using tandem dimers in which pairs of hERG subunits are expressed as single linked polypeptide chains, F656 side chains on two opposing subunits could be mutated to Ala without effect on drug block of several drugs (E-4031, terfenadine and cisapride) (Imai et al., 2009). This indicates that at least two F656 side chains can be changed to Ala without perturbing essential allosteric contributions to a high-affinity drug binding site, although for this selection of drugs, interaction with multiple F656 side chains, if present, must involve two F656 side chains on *opposite* subunits of the hERG tetrameric pore. A second possibility is that the cryo-EM structure has been caught in a conformation that is poorly compatible with high-affinity block. hERG mutations that attenuate C-type inactivation also attenuate binding of many high affinity hERG blockers, and there is circumstantial evidence that competence for inactivation in hERG is associated with repositioning of Y652 and (especially) F656 side chains into a configuration that promotes interaction with blockers in the pore (Chen et al., 2002). This might involve a small clockwise rotation of the inner S6 helix containing these side chains (Chen et al., 2002; Helliwell et al., 2018). In fact modelling suggests that the structural rearrangement of the S6 helix required to reorient F656 into a pore-facing configuration might be small (Helliwell et al., 2018), and a recent analysis of ivabradine block of hERG using molecular dynamics (MD) simulations supports some “flickering” of F656 side chains into a pore-facing configuration involving, presumably, small conformational changes in the S6 helices (Perissinotti et al., 2019). Further MD simulations should be useful in characterising conformational excursions from the cryo-EM structure that might support S6 aromatic side chain interactions with blockers in the pore. In this regard, a recent analysis of computational docking with MD simulations using the cryoEM structure to compare calculated drug binding free energies with experimental measures of drug block of hERG suggests that this approach may be useful in allowing conformational relaxation of the hERG drug binding site to maximise interactions with drugs (Negami et al., 2019). Another key piece of information might be obtained from concatemeric hERG constructs used successfully by the Sanguinetti group, that allow titration of side chain mutations separately to 1, 2, 3 or 4 subunits of the hERG tetramer (Wu et al., 2014; Wu et al., 2015); comparing block of a hERG (pseudo) tetramer containing one or two intact F656 side chains would resolve the question whether the high dependence of some blockers on F656 indicates interactions with more than one F656 side chain.

It should be said that the relationship between inactivation and high-affinity drug block in hERG is not straightforward, and the conclusion that high-affinity drugs bind more strongly to the inactivated state *per se*, is not supported by recent evidence. Sanguinetti's group demonstrated a dissociation of inactivation from high-affinity block based on drug block in hERG concatemers containing inactivation-attenuating mutations S620T or S631A in one, two, three or four subunits (Wu et al., 2015). As an example, although S620T mutation within a single subunit attenuated hERG inactivation to an extent equivalent to S620T mutation of all four subunits, the effect of these mutations on block by cisapride, dofetilide and MK499 was graded according to the number of subunits containing the S620T mutation. Measurements on a “minimally-structured” high-affinity blocker “Cavalli-2” (Cavalli et al., 2012) under conditions that promote the open, non-inactivated state or the inactivated state, respectively, showed that Cavalli-2 does not bind more strongly to the inactivated state of the wild-type channel; this is the case despite substantial reductions in Cavalli-2 block in S620T and N588K inactivation-attenuating mutants (Helliwell et al., 2018). The N588K and S631A inactivation-attenuating mutants are particularly interesting since the conformational changes in the extracellular turret of hERG (in N588K) and at the extracellular end of the selectivity filter (in S631A) (see **Figure 6**) involved in disrupting hERG inactivation must be transmitted to the cavity below the selectivity filter to disrupt binding of some high-affinity blockers. Recently Thouta et al. have argued for a dissociation of high-affinity drug block from inactivation *per se*, using a mutation, I663P, that traps hERG in an open state that nevertheless undergoes voltage-dependent inactivation (Thouta et al., 2018). They found that the extent of block of I663P hERG by terfenadine and cisapride was effectively independent of whether block was sampled following a holding potential of −80 mV (where inactivation is minimal) or +40 mV (maximal inactivation).

Recent studies using unnatural amino acid analogues that allow modulation of charge distribution within aromatic side chains supports the interpretation that interactions of drugs with canonical aromatic side chains Y652 and F656 involve aromatic stacking interactions rather than cation-π interactions (Macdonald et al., 2018), consistent with interpretations from earlier computational docking studies (Imai et al., 2009; Dempsey et al., 2014). The fact that high affinity drug blockers that bind within the hERG pore have a positive charge centre [normally a secondary, tertiary or quaternary (Perry et al., 2004; Melgari et al., 2015a) amine] is likely a result of the negative electrostatic potential below the selectivity filter resulting from focusing of the pore-helix dipole charges that is particularly strong in hERG [(Wang and MacKinnon, 2017); see **Figure 4A**]; this makes a strong contribution to the binding and location of positively-charged blockers in the hERG pore.

Support for binding of blockers within one of the hydrophobic pockets below the pore helix comes from observations that mutation of F557 on the outer pore helix (S5) to F557L (hERG F557A is poorly-expressible (Ju et al., 2009)) attenuates the effects of some hERG blockers (Saxena et al., 2016). A direct interaction between blocker and F557 would seem to require that the blocker lies deep within a hydrophobic pocket (see **Figure 4C**), and recently the serotonin receptor (5HT_1A_) agonist and dopamine (D2) receptor antagonist, sarizotan, was docked within a hydrophobic pocket in a configuration consistent with available mutagenesis data (Cheng et al., 2019). However evidence remains equivocal about direct interactions between drugs and F557; mutation of other residues that line the hydrophobic pockets (F619 and L622; see **Figure 4C**) have negligible (cisapride, haloperidol) or limited (dofetilide) effects on block by these drugs (Saxena et al., 2016), and this is surprising since computational docking indicates that drugs that bind deep within a hERG pore hydrophobic pocket are constrained within a compact binding site; as discussed below, mutations of F619 and L622 strongly attenuate the effects of a hERG activator, indicating that drug binding in this part of the channel is expected to be susceptible to mutation of these side chains. Likewise, for all drugs so far tested except cisapride, the attenuation of block in F557L is similar to the effect of the hERG Y652A mutation (Saxena et al., 2016; Helliwell et al., 2018; Cheng et al., 2019; Perissinotti et al., 2019) suggesting that the contributions of these side chains to hERG block may be linked. This interpretation is reinforced by the finding that the voltage-dependence of hERG block by Cavalli-2 is lost in hERG F557L (Helliwell et al., 2018), similar to the loss of voltage-dependence of block by this and other high-affinity blockers in hERG Y652A (Sanchez-Chapula et al., 2003). Voltage-dependence of hERG block resides in a voltage-dependent change in the configuration of the Y652 side chain resulting from interaction of the side chain phenolic OH dipole with the membrane electric field as found in other channels and receptors (Barchad-Avitzur et al., 2016). The Phe side chain cannot have intrinsic voltage responsiveness and loss of voltage-dependence of block in hERG F557L must result from an effect of the F557L mutation on a residue, or part of the channel, that *is* voltage-sensitive; a likely candidate is Y652. Wang and MacKinnon's structure provides a context for addressing these uncertainties, and a more detailed exploration of the effects of mutations of side chains that line the hydrophobic “pockets” on drug block should be productive.

The new hERG structure also provides a context for exploring the structural basis for a potential alternative access of hERG blockers to the central pore *via* the lipid bilayer, as recently proposed for the bradycardic agent ivabradine (Lees-Miller et al., 2015; Perissinotti et al., 2019). Many channel blockers, including many hERG blockers, are lipophilic and are expected to partition into the lipid bilayer phase; direct drug access from the membrane to the pore is well characterized for a number of Na_v_ (Payandeh et al., 2012) and twin-pore (K2P) channels (Dong et al., 2015), although the fenestrations required for pore access *via* the lipid are not well established in K_v_ channels (e.g. (Jorgensen et al., 2016)). In neither the hERG nor EAG structures is there a pathway that connects the central pore to the membrane that is large enough for drug molecules to enter *via* the membrane, and access *via* a lipid-facing fenestration would seem to require either a conformational state of hERG not represented in the new structures, considerable conformational flexibility or conformational transitions associated with gating. On the basis of a suppression of ivabradine block in hERG M651T and modulation of F557 and F656 interactions by M651 in MD simulations (see **Figure 4C** for the location of these residues) it is proposed that these three residues act as a dynamic gate to facilitate ivabradine access from the membrane to the inner pore (Perissinotti et al., 2019); however, direct evidence from experiment or simulation for access to the pore [the likely site of ivabradine channel block (Melgari et al., 2015b; Perissinotti et al., 2019)] from the membrane remains to be established.

## The hERG Structure and hERG Activators

**Figure 5:**
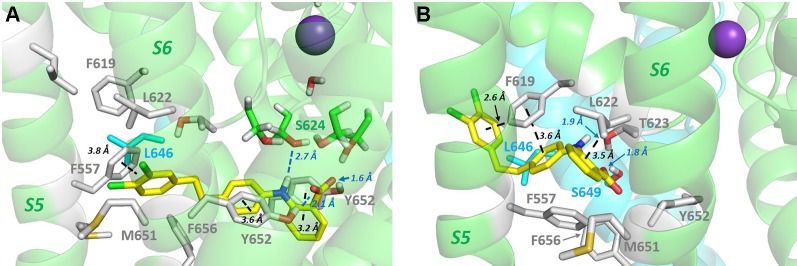
**(A)** PD-118057 (yellow sticks) can be docked into the hERG structure in configurations that orient the benzyl carboxyl group to interact with K^+^ ions as they traverse the hERG conductance pathway as suggested in (Schewe et al., 2019). However in these configurations PD-118057 does not make favourable interactions with F619, L622 (and L646 on the adjacent subunit), identified as key binding determinants for this activator (Perry et al., 2009). Also PD-118057 would be expected to interact with Y652 and F557 in these states whereas mutagenesis of Y652 and F557 has minimal effect on activator binding (Perry et al., 2009). **(B)** Docked states consistent with mutagenesis (Perry et al., 2009) (aromatic stacking and van der Waals interactions with F619, L622 and adjacent L646 side chain) can be found deeper within the hydrophobic pockets below the pore helix, but these states are not compatible with interaction of the PD-118057 carboxylate with K^+^ ions in the pore as suggested in (Schewe et al., 2019). To orient the viewer, PD-118057 occupies a hydrophobic pocket similar to that shown in **Figure 4C** for Cavalli-2 binding, with the dichlorophenyl group (chlorine atoms green) of PD-118057 close to the membrane in panel B. Docking was performed with GOLD version 5.6; Cambridge Crystallographic Data Centre, Cambridge, UK as described previously (Dempsey et al., 2014; Helliwell et al., 2018).

In addition to drug block susceptibility, hERG is activated by sets of molecules that act by attenuating inactivation and/or shifting its voltage-dependence to depolarizing potentials, enhancing K^+^ conductance or slowing channel deactivation (or some combination of these) (Sanguinetti, 2014). hERG activators have promise in treating cardiac disorders involving hERG dysfunction, for example the subset of long QT syndrome cases arising from dysfunction of mutant channels that are trafficked to the plasma membrane (Grunnet, 2010; Anderson et al., 2014). One set of activators that comprises molecules containing a negatively-charged group, exemplified in the case of hERG by the activator PD-118057, has recently been shown to interact promiscuously across a range of twin pore and voltage-sensitive K^+^ channels (including hERG), to bind below the selectivity filter, and suggested to promote K^+^ occupancy of sites within and below the selectivity filter, enhancing channel conductance (Schewe et al., 2019). PD-118057 was previously shown using mutagenesis to interact with the hERG pore helix: PD-118057 activation of hERG was effectively eliminated in hERG F619A, and L622C strongly attenuated activation (Perry et al., 2009) (see **Figures 4C** and **5** for the location of these residues). These studies provide strong evidence that PD-118057 (and possibly other members of this class of negatively-charged activators) occupy one or more (Wu et al., 2014) of the hydrophobic pockets. Accordingly, it is possible to find low energy score docked configurations for PD-118057 bound within a hydrophobic pocket with the negative carboxylate group oriented to interact with K^+^ ions as they traverse the hERG pore and enter the selectivity filter (**Figure 5A**). However, while these configurations appear consistent with interpretations from the structural analysis of negatively-charged activators with twin pore K^+^ channels (Schewe et al., 2019), they match less well with the earlier mutagenesis data (Perry et al., 2009) that support a binding of PD-118057 deeper within a pocket involving the pore helix and residues on the S5-S6 interface (**Figure 5B**). **Figure 5** illustrates what is essentially a geometric argument that, at least in the context of the new cryo-EM structure, binding of PD-118057 within a hydrophobic pocket with its carboxylate oriented to interact with K^+^ ions in the pore would seem to require interaction with residues whose mutations have minimal effect on binding [especially Y652; also F557 (Perry et al., 2009)], while interacting less well with the residues deeper in the pocket that are critical for binding [F619, L622 and L646 of an adjacent subunit (Perry et al., 2009)].

In addition, while Schewe et al. propose an enhancement of channel conductance arising from negative charge promotion of occupation of K^+^ sites in or below the selectivity filter as a mechanism for the activation of TREK channels by negatively charged activators (Schewe et al., 2019), Perry et al. showed that PD-118057 does not promote single channel conductance of hERG, and that it acts largely by attenuating inactivation (increasing channel open probability, P_o_), most likely by suppressing conformational changes involving the pore helix that are involved in hERG inactivation (Perry et al., 2009). Interestingly, PD-118057 antagonises the blocking effect of at least one potent hERG blocker, terfenadine, and while this may suggest overlapping binding sites between activator and blocker (Schewe et al., 2019), an alternative interpretation is that the conformational changes associated with hERG inactivation that are suppressed by PD-118057 are also linked to optimisation of the configuration of side chains on the S6 helix (Y652 and F656) for interaction with blockers in the channel pore (Chen et al., 2002; Helliwell et al., 2018) as discussed above.

LUF7244 is another hERG activator reported also to act by attenuating rapid inactivation (Qile et al., 2019), that suppresses binding of hERG inhibitors (cisapride, astemizole, dofetililde and sertindole (Yu et al., 2016)). The potential of hERG activators as therapeutics to counteract the effects of drug-induced LQTS is shown by the recent demonstration of suppression of dofetilide-induced *torsades de pointes* arrhythmia in a dog model (Qile et al., 2019) by this activator. LUF7244 can be docked into the hERG pore domain of the cryo-EM structure between two subunits and partly occupying a hydrophobic pocket in a manner that might overlap with the binding site for dofetilide (Qile et al., 2019) which is expected to lie largely in the K^+^ permeation pathway below the selectivity filter (Kamiya et al., 2006). On the other hand, [^3^H]dofetilide binding displacement by LUF7244 indicates that this activator is an allosteric modulator of dofetilide binding with a distinct (non-overlapping) binding site (Yu et al., 2016), and so this activator might constitute an additional example where the suppression of inactivation also suppresses inactivation-associated conformational changes below the selectivity filter that optimise high-affinity inhibitor binding. Consideration of both inhibitor and activator binding therefore suggests that the hERG cryo-EM structure may have been captured in a pre-inactivated state which is optimal for binding of the class of activators that suppress conformational transitions that are involved in inactivation and which promote the binding of some high-affinity pore blockers. The new structure provides a context for addressing the interplay between activator and inhibitor binding in this class of hERG activators, and this should facilitate further development of activators having therapeutic potential.

## The hERG Structure and Channel Gating

**Figure 6:**
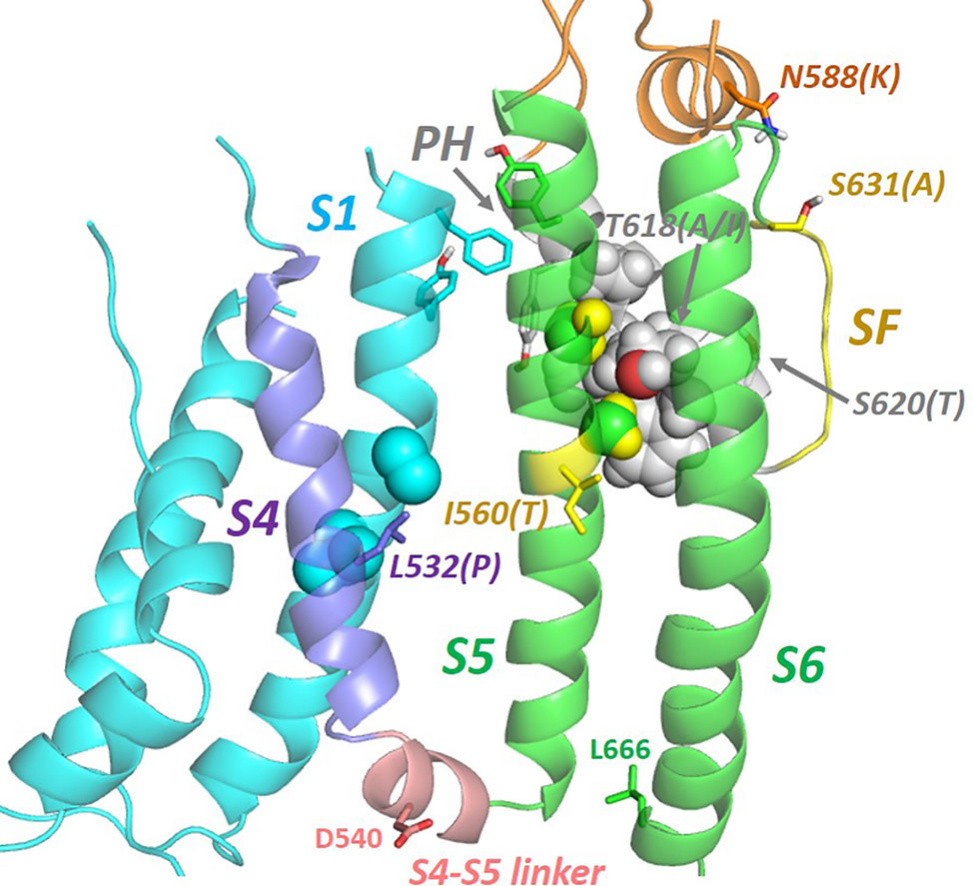
Natural mutations (in brackets) in hERG that perturb inactivation gating are found throughout the membrane domain indicating a network of helix interactions that transmits conformational changes to the selectivity filter (SF) resulting from mutation or voltage sensor (VS) activation. In the VS-activated state captured in the cryo-EM structure, residues on S4 whose mutation perturbs inactivation [e.g. L532P; (Hassel et al., 2008; Zhang et al., 2011)] interact closely with side chains of the S1 helix. The extracytoplasmic ends of the S1, S5 and pore helices interact *via* a cluster of aromatic side chains (see also **Figure 7**). S5 and pore helix side chains interdigitate (‘knobs into holes' packing) indicating a strong conformational coupling of these helices. The locations of D540 and L666 that move close together upon membrane repolarization are shown [adapted from (Butler et al., 2019)].

Two features of hERG gating are of particular interest: (i) the manner in which voltage-linked conformational changes in the voltage sensor (VS) domain couple to the activation gate, and (ii) the structural rearrangements that lead to the rapid C-type inactivation that is a distinguishing feature of hERG. In the first case the cryo-EM structure of hERG (and the earlier structure of rEAG) provide the structural context for rationalizing the curious observations that splitting of hERG (Lorinczi et al., 2015; de la Pena et al., 2018) and EAG (Lorinczi et al., 2015; Tomczak et al., 2017; Malak et al., 2019) in the S4–S5 linker between voltage sensor and pore domains yields channels that open and close in response to changes in membrane potential much like the wild type channels; other distinguishing features of these channels (e.g. inward rectification/rapid inactivation in hERG; modulation of EAG activation time constant by external Mg^2+^ and prepulse potential) are broadly retained in the split channels (Lorinczi et al., 2015). In both hERG and EAG the non-domain-swapped organization and packing of VS domains against their own pore subunits (see **Figure 2**) is associated with a very short S4–S5 linker sequence (Whicher and MacKinnon, 2016; Wang and MacKinnon, 2017) (**Figure 6**), and recent studies support one of two possible mechanisms for VS-mediated closing of the hERG activation gate at polarizing potentials that accommodates retention of gating in split channels: (i) the S4–S5 linker acts as a ligand that binds to the C-terminal end of the inner pore S6 helix below the activation gate, locking the channel in a closed state. Upon depolarization upward movement of the S4 helix pulls the linker out of its binding site on S6 allowing the pore to open (Malak et al., 2017; Malak et al., 2019). (ii) The C-terminal end of the S4 helix “pushes” on the bottom of the S6 helix in the membrane polarized state closing the activation gate, and this pushing is relieved by upward movement of S4 upon membrane depolarization (Tomczak et al., 2017; de la Pena et al., 2018). These mechanisms are similar in that they involve interaction of elements at the bottom of the VS S4 helix and/or S4–S5 linker with the C-terminal end of the S6 helix (Tristani-Firouzi et al., 2002), and are consistent with evidence that the hERG pore is “naturally” open in the absence of a membrane potential (e.g. (Tristani-Firouzi et al., 2002)). In the cryo-EM structure the VSD is in an activated configuration consistent with a depolarized membrane potential (*i.e.* 0 mV in the conditions of cryo-EM), with an open activation gate resulting from releasing the constraints of S4 on the bottom of the S6 helix that are described above. The S4 helix is therefore in an “upward” configuration with the two S4 arginine side chains identified as carrying gating charge (Arg 528 and Arg 531) (Zhang et al., 2004) lying above the hydrophobic “plug” (Cheng et al., 2013) that separates the intra-and extracellular hydrated segments of the VSD. The structure of the VSD at repolarized potentials that involves reconfiguration of S4 into a “downward” state is so far structurally undefined. However, the fact that D540C in the short S4–S5 linker forms a disulphide bond with L666C at the bottom of S6 in the hERG D540C/L666C double mutant that locks the activation gate closed indicates that these residues (D540 and L666) are close together upon membrane repolarization (Ferrer et al., 2006) (see **Figure 6**).

The most prominent feature of hERG gating is the rapid onset of C-type inactivation at depolarized potentials, followed by recovery from inactivation during the repolarization phase of the action potential (see Sanguinetti and Tristani-Firouzi, 2006; Vandenberg et al., 2012) for detailed descriptions of the hERG gating cycle). This property combined with a slow channel deactivation results in an efficient return to the cardiac myocyte resting membrane potential. Like other K^+^ channels hERG inactivation involves loss of K^+^ occupation of one or more sites within the selectivity filter (Vandenberg et al., 2012; Armstrong and Hollingworth, 2018). Comparison of the selectivity filter in the hERG structure with that of EAG (which doesn't inactivate) suggests that a ring of hydrogen bonding side chains involving S631 and N629 on adjacent subunits encircles the top of the selectivity filter (which comprises the sequence S_624_VGFG_628_) and might act as a compressible “spring” to modulate access and occupancy of the outer site(s) of the selectivity filter (see **Figure 2A**), an interpretation that is supported by an apparent change in mode of binding of the scorpion toxin CnErg1 to directly “plug” the selectivity filter in hERG S631A (Hill et al., 2007). Replacing hERG S631 with alanine (in hERG S631A) to match the equivalent EAG sequence was shown many years ago to result in loss of inactivation (Schonherr and Heinemann, 1996); this mutation has recently been found as a rare natural hERG variant with a clinical manifestation of SQTS (Akdis et al., 2018). The hERG cryo-EM structure is particularly valuable in addressing the structural context of natural channel mutations that underlie hERG-related channelopathies and the potential this may afford for optimising therapeutic intervention with hERG blocking antiarrhythmic agents (in the case of SQTS (Hancox et al., 2018)) or activators in the case of LQTS (Sanguinetti, 2014). Thus recent electrophysiological characterization of the S631A mutation indicates that hERG blockers like quinidine, with low dependence on inactivation (lacking in hERG S631A), may be effective in suppressing premature repolarization in this class of SQTS mutants (Butler et al., 2018).

In the light of these observations, the cryo-EM structure of the hERG_TS_ S631A mutant construct published with the hERG_T_ structure (Wang and MacKinnon, 2017) is both fascinating and puzzling. The S631A mutant structure differs from the hERG_T_ (and hERG_TS_) structure only by a small shift in the position of the side chain of F627, which lies behind the selectivity filter, to a configuration that closely matches the configuration of the equivalent aromatic side chain in other K_v_ channels (e.g. EAG, KcsA and K_v_Chim; see (Wang and MacKinnon, 2017)). In other words the position of F627 in hERG_T_ and hERG_TS_ is anomalous in the context of other K_v_ channels (and hERG_TS_ S631A) that have more limited inactivation properties, leading to the suggestion that hERG_T_ (and hERG_TS_) represents an inactivated state as would be expected in the absence of a membrane potential (i.e. at 0 mV, well above the V_0.5_ for inactivation in hERG) (Wang and MacKinnon, 2017). However, this seems contrary to the expectations that inactivation in hERG is associated (i) with conformational changes that are transmitted to the region of the pore domain below the selectivity filter as described earlier, and (ii) conformational changes involving the hERG turret helix as described below. MD simulations may be useful in establishing whether the configuration of F627 in the hERG structure results in sufficient perturbation of the selectivity filter to attenuate K^+^ ion occupancy as expected for an inactivated state. If this is the case then the more extensive conformational changes expected to be associated with hERG inactivation might be subsequent events on the inactivation pathway [see, e.g., (Wang et al., 2011)] that are not captured in the cryo-EM structure.

The hERG structure also provides a context for exploring the pathway(s) by which inactivation-gating develops upon voltage-sensor activation, and again the study of natural inactivation-perturbing hERG mutants is proving useful. Recent consideration of the structural context of the inactivation-attenuating I560T mutation that lies on the outer pore (S5) helix suggests that conformational perturbation of the S5 helix is likely to be linked to the selectivity filter *via* intimate interactions with the pore helix (**Figure 6**) (Butler et al., 2019). In fact, inspection of several natural inactivation-perturbing hERG mutations suggests that their effects are transferred to the selectivity filter through a network of helix–helix interactions involving the S4 and S1 helices of the voltage sensor domain and the S5 and pore helices of the pore domain (**Figure 6**) (Butler et al., 2019). This interpretation differs somewhat from an inactivation pathway previously proposed that involves direct interaction between the voltage sensor S4 helix and the S5 helix (Perry et al., 2013). Although this is incompatible with the cryo-EM structure, S4–S5 interactions might occur in another gated state (e.g. the deactivated state at resting membrane potentials). As described above, D540C forms a disulphide bond with L666C in the hERG D540C/L666C double mutant; since the S4–S5 linker is short, apposition of D540 and L666 would seem to require a close approach of S4 and S5 at least at the cytoplasmic end of these helices when membrane repolarization reconfigures the VS domain to close the activation gate (see **Figure 6**).

## A “Tryptophan Clamp” Linking the S5 and Turret Helices in KCNH Channels?

**Figure 7:**
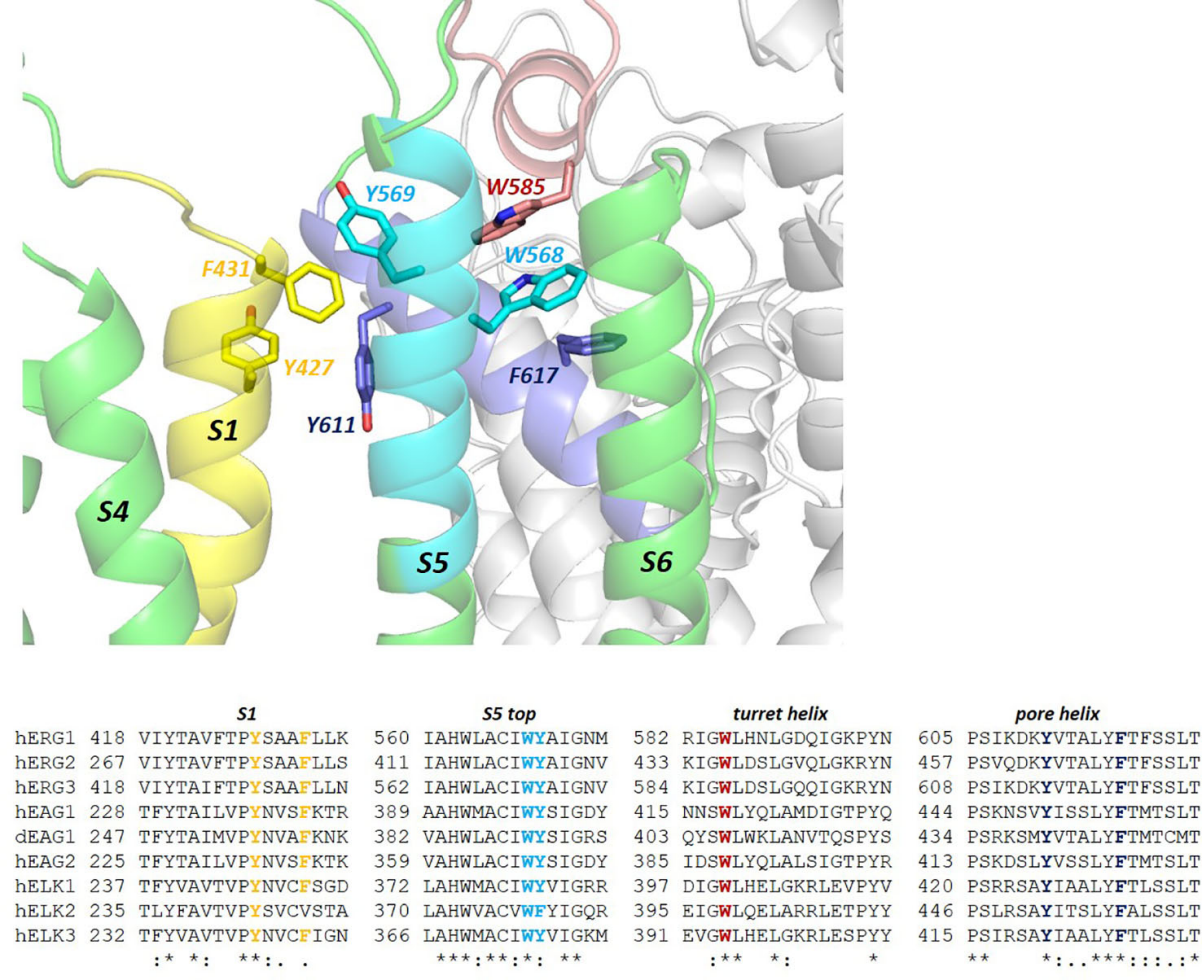
A “tryptophan clamp” connects the hERG turret helix with the top of the S5 helix. This motif that includes an interaction with F617 on the hERG pore helix is conserved throughout the KCNH family of channels that includes ERG, EAG and ELK variants, despite considerable sequence diversity in the turret helix itself (G584-I593 in hERG1). Helix interactions involving a cluster of aromatic side chains at the extracytoplasmic ends of S1, S5 and the pore helix may serve to anchor the VS domain against the pore as observed in other K_v_ channels (Lee et al., 2009); dEAG1 is drosophila EAG1; h, human.

A distinguishing feature of KCNH channels including the hERG (*KCNH2*) and rEAG (*KCNH1*) variants for which structures are now available (Whicher and MacKinnon, 2016; Wang and MacKinnon, 2017), is a long amino acid sequence that links the C-terminal end of the S5 helix and the N-terminal end of the pore helix on the extracellular side of the channel (see **Figures 1** and **2**). Evidence that this “turret” region of KCNH channels contains an amphipathic helix (Liu et al., 2002; Torres et al., 2003) is confirmed in both the hERG (**Figures 1** and **2A**) and rEAG structures; moreover the structures suggest how conformational changes in the S5, pore helix and turret helices may be connected to facilitate inactivation in hERG. The S5 and turret helices are linked by pair of a stacked tryptophan side chains (a “tryptophan clamp”) that interacts with a phenylalanine side chain on the pore helix (**Figure 7**). This arrangement is conserved, at least in amino acid sequence, across the KCNH family (that also includes ELK channels) (**Figure 7**), and is reminiscent of a similar structural motif that mediates dimerization in the ligand binding domain of the human pregnane X receptor (PXR) (Noble et al., 2006).

In hERG the turret helix is involved in fast C-type inactivation, and mutations on the polar face of the turret helix can attenuate [e.g. N588K (Brugada et al., 2004)] or enhance (e.g. N588E) inactivation by shifting the voltage-dependence to depolarizing or polarizing potentials, respectively (Clarke et al., 2006). The tryptophan clamp likely provides a structural link between the turret and the rest of the channel that transmits conformational changes involving S5 and the pore helix, to the turret helix. In hERG this leads to conformational changes that underlie fast inactivation, and these are expected to involve reorientation of the turret helix that allows residues on its polar face (N588 and Q592 in hERG) to interact with polar groups on the top of the selectivity filter (e.g. backbone and/or side chains of N629, S631, N633 and T634 in hERG; see **Figure 2**). hEAG1 doesn't inactivate; however a chimera with the S5P linker of EAG transplanted into hERG inactivates with characteristics similar to hERG itself (Herzberg et al., 1998), and inactivation can be recovered in EAG1 by mutating residues equivalent to S620 and S631 in hERG (T432 and A443 in bovine EAG1) to serine (Ficker et al., 2001), indicating that the turret helix in EAG likely acts in an equivalent manner to the hERG turret helix. Likewise, although WT human (and bovine) EAG1 doesn't inactivate, the drosophila version of EAG1 (dEAG1) does inactivate (Robertson et al., 1996); surprisingly, dEAG1 has a lysine in the equivalent position of the turret helix as N588 in hERG (K409 in dEAG), substitution of which to lysine in hERG (N588K; see **Figure 6**) strongly *attenuates* inactivation. Interestingly, dEAG1 has an E462 at the homologous position as N633 and, as indicated above, these residues at the top of the selectivity filter may provide interacting “partners” for complementary residues on the polar face of the turret helix when gating-induced conformational changes move the turret helix closer to the top of the selectivity filter as suggested from intersubunit disulphide bond formation in turret helix cysteine mutants (Jiang et al., 2005). Altogether, these observations support the conclusion that the turret helix responds similarly across the KCNH family to gating-induced conformational changes elsewhere in the protein [rELK2 rapidly inactivates when its turret sequence is replaced by that of hERG (Clarke et al., 2006)], with the specific modulating effect on gating (e.g. rapid inactivation in hERG) depending on the particular amino acid sequence of the turret helix and the amino acids near the top of the selectivity filter that may interact with the turret at specific depolarizing potentials. The new structures for hERG and rEAG provide the structural context to explore and understand these gating modulating mechanisms.

## Conclusions

Consideration of the cryo-EM structure of hERG in the context of a large body of work obtained both before and following its publication suggests that channel may have been captured in a pre-inactivated open state, despite the expectation that hERG should be largely inactivated at a membrane potential of zero mV. Although this complicates understanding of the manner in which high affinity drugs interact with the channel (since some of these require conformational changes associated with “inactivation-competence” for maximal binding affinity), it suggests that the structure may be a good template for optimising the design of the class of hERG activators that suppress inactivation by binding and stabilising pre-inactivated open states. The hERG and rEAG structures should also be useful templates for molecular dynamics (MD) simulations to explore the local structural and dynamic effects of natural mutations that affect gating and the conformational changes involving linked reconfiguration of the S5, pore- and turret-helices that modulate gating in the KCNH family. As usual, it would be desirable to have more structural information, and a wish-list would include hERG structures complexed with a high affinity blocker, with an inactivation-attenuating activator and a structure trapped in a deactivated (resting) state. The latter is notoriously problematic for voltage sensitive ion channels since these channels have activated voltage sensors in the absence of a membrane potential. In the case of hERG the D540C, L666C double mutant with a disulphide bond between the S4–S5 (D540C) and S6 (L666C) helices might be a useful target for structural analysis since this mutant is trapped in a closed state with the voltage sensor presumably in its non-activated state (Ferrer et al., 2006). However, for now the cryoEM structure for hERG is providing a fascinating structural context for exploring and interpreting a wealth of experimental data on this biophysically and pharmacologically important channel.

## Author Contributions

CD and JH wrote the manuscript with the input of all the authors. AB, MH, and YZ contributed information on hERG blockers and natural hERG mutants, and YZ prepared **Table 1**.

## Funding

We acknowledge funding from the British Heart Foundation for support of MVH (FS/14/38/30868) and YZ (PG/17/89/33414).

## Conflict of Interest

The authors declare that the research was conducted in the absence of any commercial or financial relationships that could be construed as a potential conflict of interest.
